# Functional Recovery Trajectory of Temporomandibular Joint Following Condylar Fracture Treated by Closed Reduction and Intermittent Jaw Mobilization: A Longitudinal Assessment Using Helkimo’s Clinical Dysfunction Index

**DOI:** 10.7759/cureus.117381

**Published:** 2026-10-03

**Authors:** Kiruthika Babu, Ajith Kumar K, Sooraj KS, Sherin Rahim, Ilavarasan A

**Affiliations:** 1 Department of Oral and Maxillofacial Surgery, Government Dental College, Thiruvananthapuram, IND; 2 Department of Prosthodontics, Government Dental College, Thiruvananthapuram, IND

**Keywords:** closed fracture reduction, condyle fracture, craniofacial trauma, helkimo’s index, temporomandibular joint

## Abstract

Background

The optimal treatment of mandibular condylar fractures remains controversial, as comparative studies have demonstrated variable outcomes following open reduction and internal fixation and closed treatment, with the available evidence not establishing a universally superior treatment modality. Closed reduction of condylar fractures aims to allow fracture healing, taking the patient's occlusion as a reference for fracture management. Early functional mobilization has been advocated following condylar fractures because prolonged immobilization may contribute to joint stiffness, fibrous adhesions, and restricted mandibular movement, potentially increasing the risk of post-traumatic ankylosis. Hence, intermittent jaw mobilization has been instituted to maintain functional mandibular movement during the healing period and to minimize prolonged immobilization of the temporomandibular joint (TMJ). However, the functional recovery trajectory of the joint after fracture of the condyle follows a unique trend, and this study aims to map this trajectory of fracture healing to functional healing of the joint following condylar fractures.

Methods

A prospective longitudinal observational study was conducted among 63 patients presenting with mandibular condylar fractures and managed conservatively by closed treatment with an intermittent jaw mobilization protocol. The mapping of functional recovery of the TMJ was assessed at the first visit, at six weeks, and at three months, and the assessment was done using Helkimo’s Clinical Dysfunction Index.

Results

The longitudinal assessment in the present study provides a more meaningful representation of functional recovery than a single postoperative assessment. The observed trajectory highlights that clinical functional recovery may evolve progressively over time; however, structural healing or morphological changes of the condyle were not directly assessed radiographically in the present study. Therefore, the observed data demonstrate temporal improvement in TMJ function following conservative treatment with intermittent mobilization, but due to the observational design and absence of a comparison group, the observed improvement in functional parameters cannot be interpreted as evidence of treatment effectiveness or causality.

## Introduction

Mandibular condylar fractures are frequently associated with alterations in mandibular movement and temporomandibular joint (TMJ) function. Although closed treatment with early, intermittent jaw mobilization is commonly used, the pattern and rate of functional recovery may vary among patients and may continue over the course of follow-up. Therefore, longitudinal clinical assessment is important for documenting changes in mandibular function and identifying persistent dysfunction following condylar fractures. Helkimo’s Clinical Dysfunction Index provides a standardized clinical assessment of TMJ dysfunction based on five domains: limitation of mandibular mobility, impaired function of the masticatory system, pain during mandibular movement or palpation, tenderness of the masticatory muscles and TMJ, and TMJ sounds [1]. Each domain is clinically graded according to predefined criteria, allowing the overall severity of clinical dysfunction to be categorized systematically.

The present study aims to describe the longitudinal trajectory of functional recovery following closed treatment with intermittent jaw mobilization in patients with mandibular condylar fractures using Helkimo’s Clinical Dysfunction Index.

## Materials and methods

Aim

To longitudinally evaluate the trajectory of functional recovery following closed treatment and intermittent jaw mobilization of mandibular condylar fractures using Helkimo’s Clinical Dysfunction Index.

Study design and setting

A prospective longitudinal observational study was conducted to evaluate the trajectory of functional recovery following conservative management of mandibular condylar fractures using a standardized intermittent jaw mobilization protocol. The study included 63 patients diagnosed with mandibular condylar fractures and considered suitable for closed treatment. The study was conducted in the Department of Oral and Maxillofacial Surgery, Government Dental College, Thiruvananthapuram, Kerala, India, following approval from the Institutional Ethics Committee (Approval No.: IEC/E/08/2020/GDCT, dated December 17, 2020). All participants were recruited after obtaining written informed consent.

The study was designed to assess changes in TMJ function and mandibular mobility longitudinally at predefined follow-up intervals. Particular emphasis was placed on the change in Helkimo’s Clinical Dysfunction Index as a measure of functional recovery over time [1].

Study population 

Patients presenting to the Department of Oral and Maxillofacial Surgery with clinically and radiographically diagnosed mandibular condylar fractures were screened for eligibility. The diagnosis and characterization of the condylar fracture were established using a combination of clinical examination and appropriate radiographic investigation using computerized tomography scans. 

Baseline clinical and radiographic assessment

At the initial assessment, demographic and clinical characteristics were recorded using a standardized data collection format. The variables recorded included age, sex, mechanism of injury, side of involvement, and relevant associated injuries. Radiographic evaluation was performed to confirm the diagnosis and characterize the fracture to determine the suitability of choosing patients for conservative management [2]. Radiographic healing was not used as an outcome measure, and no serial radiographic assessment of fracture healing was performed in this study. The anatomical level of the condylar fracture, unilateral or bilateral fracture status, degree and direction of displacement, and presence or absence of dislocation were documented based on Lindahl classification [3,4] in order to exclude patients indicated for surgical management from this study.

Inclusion criteria

Patients diagnosed with unilateral or bilateral intracapsular condylar fractures considered suitable for closed treatment, who were capable of performing active mandibular exercises and willing and able to comply with the prescribed jaw mobilization and follow-up protocol and provided written informed consent for participation in the study.

Exclusion criteria

Condylar fractures requiring open reduction and internal fixation as per Zide and Kent criteria [2], previous history of TMJ disorders or significant pre-existing mandibular dysfunction, previous mandibular or TMJ surgery, associated mandibular fractures requiring treatment that could independently influence mandibular movement or occlusion, neurological or musculoskeletal conditions affecting mandibular movement.

Assessment of mandibular function

Functional assessment was performed using Helkimo’s Clinical Dysfunction Index. The index was selected as the principal functional outcome measure because it provides a standardized clinical assessment of mandibular dysfunction.

The Clinical Dysfunction Index evaluates five principal domains of mandibular dysfunction: (1) Impairment of mandibular mobility; (2) Impaired function of the temporomandibular system; (3) Pain during mandibular movement; (4) TMJ and masticatory muscle tenderness; and (5) TMJ sounds.

Each component was scored according to the established Helkimo scoring system (Table 1) [1], and the individual scores were summed to obtain the overall Helkimo’s Clinical Dysfunction Index (Di) score.

**Table 1:** Helkimo’s Clinical Dysfunction Index (Di) TMJ: temporomandibular joint

Component	Clinical finding	Score
A. Impaired mandibular mobility	Maximum mouth opening > 40 mm | Maximum mouth opening 30-39 mm | Maximum mouth opening <30 mm Maximum lateral movement > 7 mm (Right/Left) | Maximum lateral movement 4-6 mm (Right/Left) | Maximum lateral movement 0-3 mm (Right/Left) Maximum protrusion >7 mm | Maximum protrusion 4-6 mm | Maximum protrusion 0-3 mm	0,1,5
Sum = 0 - Normal Mobility (A=0) Sum = 1-4 - Slight impaired mobility (A=1) Sum = 5-20 - Severe impaired mobility (A=5)		
B. Impaired TMJ function	Smooth movement without TMJ sounds and deviation ≤2 mm | TMJ sounds in one/both joints and/or deviation >2 mm | Locking and/or luxation of TMJ	0,1,5
C. Muscle pain	No tenderness on palpation of masticatory muscles | Tenderness at 1–3 palpation sites | Tenderness at ≥4 palpation sites	0,1,5
D. TMJ pain	No tenderness on palpation | Tenderness on lateral palpation | Tenderness on posterior palpation	0,1,5
E. Pain on mandibular movement	No pain during mandibular movement| Pain during one movement | Pain during ≥2 movements	0,1,5

The corresponding interpretation of the Di score was recorded according to the validated classification system presented in Table 2.

**Table 2:** Interpretation Di score: Helkimo’s Clinical Dysfunction Index score

Total Di score	Classification	Interpretation
0	Di 0	No clinical dysfunction
1-4	Di I	Mild clinical dysfunction
5-9	Di II	Moderate clinical dysfunction
10-25	Di III	Severe clinical dysfunction

The Di score was recorded at each predefined assessment point, allowing longitudinal comparison of functional status and assessment of the trajectory of recovery following conservative treatment.

Conservative treatment and intermittent jaw mobilization protocol

All patients included in the study were managed using a standardized closed-treatment protocol [5]. Following initial management of the fracture, upper and lower arch bars were placed, and inter-maxillary fixation/elastics were used to maintain the achieved occlusion. After satisfactory occlusion was established, patients were managed according to an intermittent jaw mobilization protocol. In this protocol, intermittent release of the fixation was initiated at the end of every week, and active mandibular mobilization was commenced [6]. During the active mobilization phase, patients were instructed and supervised regarding active mandibular exercises. The exercise regimen included active mouth opening, right and left lateral mandibular excursions, and protrusive mandibular movements. Patients were instructed to perform the prescribed exercises in a controlled and progressive manner with repetitions of each movement for approximately one hour. The prescribed mandibular exercises were single examiner-supervised sessions and were not prescribed as home exercises. Therefore, a separate home-exercise compliance record or patient-maintained exercise diary was not used.

In every visit, the patient’s occlusal stability following mandibular mobilization was assessed. This was taken as a guiding parameter to determine the endpoint of elastic traction and inter-maxillary fixation. The intermittent mobilization phase was continued till the definitive removal of inter-maxillary fixation/elastics after occlusal stability was achieved. This intermittent mandibular mobilization protocol was followed for both unilateral and bilateral condylar fractures that were indicated for closed reduction and management.

Follow-up assessment

Patients were evaluated longitudinally at three predefined assessment points: (1) Baseline/first assessment; (2) Six-week assessment; and (3) Three-month assessment.

At each assessment, the clinical evaluation was repeated using the same assessment parameters. Helkimo’s Clinical Dysfunction Index was reassessed, and objective mandibular movement parameters-including maximum interincisal opening, right and left lateral excursion, protrusion, and deviation on opening-were recorded.

Study outcomes

The primary outcome of the study was the longitudinal change in Helkimo’s Clinical Dysfunction Index from baseline through the six-week and three-month follow-up assessments. The pattern of change in Di scores across these time points was used to characterize the functional recovery trajectory following conservative treatment of condylar fractures.

The serial assessment of these parameters enabled evaluation of whether functional recovery occurred progressively during the early rehabilitation period and whether improvement was maintained or further enhanced at the three-month assessment.

Data collection and statistical analysis

Statistical analysis was performed to evaluate the longitudinal change in mandibular functional status following conservative management of mandibular condylar fractures. The analysis included a total of 63 patients, and all 63 participants completed the scheduled functional assessments at baseline, six weeks, and three months. Therefore, there were no losses to follow-up and no missing functional outcome assessments among the enrolled study participants.

Continuous variables were summarized using mean ± standard deviation (SD), median, and range, while categorical variables were expressed as frequencies and percentages. The distribution of continuous variables was assessed using the Shapiro-Wilk test. The overall change in Di score across baseline, six weeks, and three months was assessed using the Friedman test (Table 3). Where a statistically significant overall difference was identified, pairwise post-hoc comparisons between baseline and six weeks, baseline and three months, and six weeks and three months were performed using the Wilcoxon signed-rank test.

**Table 3:** Statistical summary table TMJ: temporomandibular joint; IQR: interquartile range

Parameter	First visit	Six-week follow-up	Three-month follow-up	Statistical test and p-value
TMJ pain/muscle pain/pain on mandibular movement/TMJ sounds	Mean +/- SD (or Median (IQR))	Mean +/- SD (or Median (IQR))	Mean +/- SD (or Median (IQR))	ANOVA/Friedman test (p < 0.05)
Mandibular mobility score	Mean +/- SD/Median (IQR)	Mean +/- SD/Median (IQR)	Mean +/- SD/Median (IQR)	Friedman test (p < 0.05)

Because three pairwise comparisons were performed, the Bonferroni correction was applied to control for the family-wise type I error. The Bonferroni-adjusted level of statistical significance was calculated using the formula:

$\alpha_{\mathrm{adjusted}} = \frac{\alpha}{n}$

where $\alpha_{\mathrm{adjusted}}$ is the adjusted level of statistical significance, *α* is the original significance level, and *n* is the number of statistical comparisons.

For the present analysis:

$\alpha_{\mathrm{adjusted}} = \frac{0.05}{3} = 0.0167$

Accordingly, a two-tailed p-value <0.0167 was considered statistically significant for the post-hoc pairwise comparisons.

The Di scores were additionally categorized according to the established clinical dysfunction grading system as no dysfunction, mild dysfunction, moderate dysfunction, and severe dysfunction. Changes in the distribution of patients across these categories at baseline, six weeks, and three months were used to describe the clinical pattern of functional recovery. For each longitudinal comparison, the corresponding test statistic and p-value were reported. The Friedman test was expressed using the χ² test statistic, whereas the Wilcoxon signed-rank test was expressed using the W statistic and corresponding standardized Z value where applicable.

All statistical tests were two-tailed. A p-value <0.05 was considered statistically significant for the primary overall analysis, while the Bonferroni-adjusted threshold of p<0.0167 was applied for the three post-hoc pairwise comparisons. A 95% confidence level was used throughout the analysis.

## Results

Data interpretation

The longitudinal assessment demonstrated a clear trend toward progressive improvement in clinical dysfunction following closed reduction management of condylar fracture. At the initial assessment (Figure 1), the majority of patients exhibited moderate-to-severe dysfunction, with severe dysfunction (n=34,53.2%) being the predominant category.

**Figure 1:**
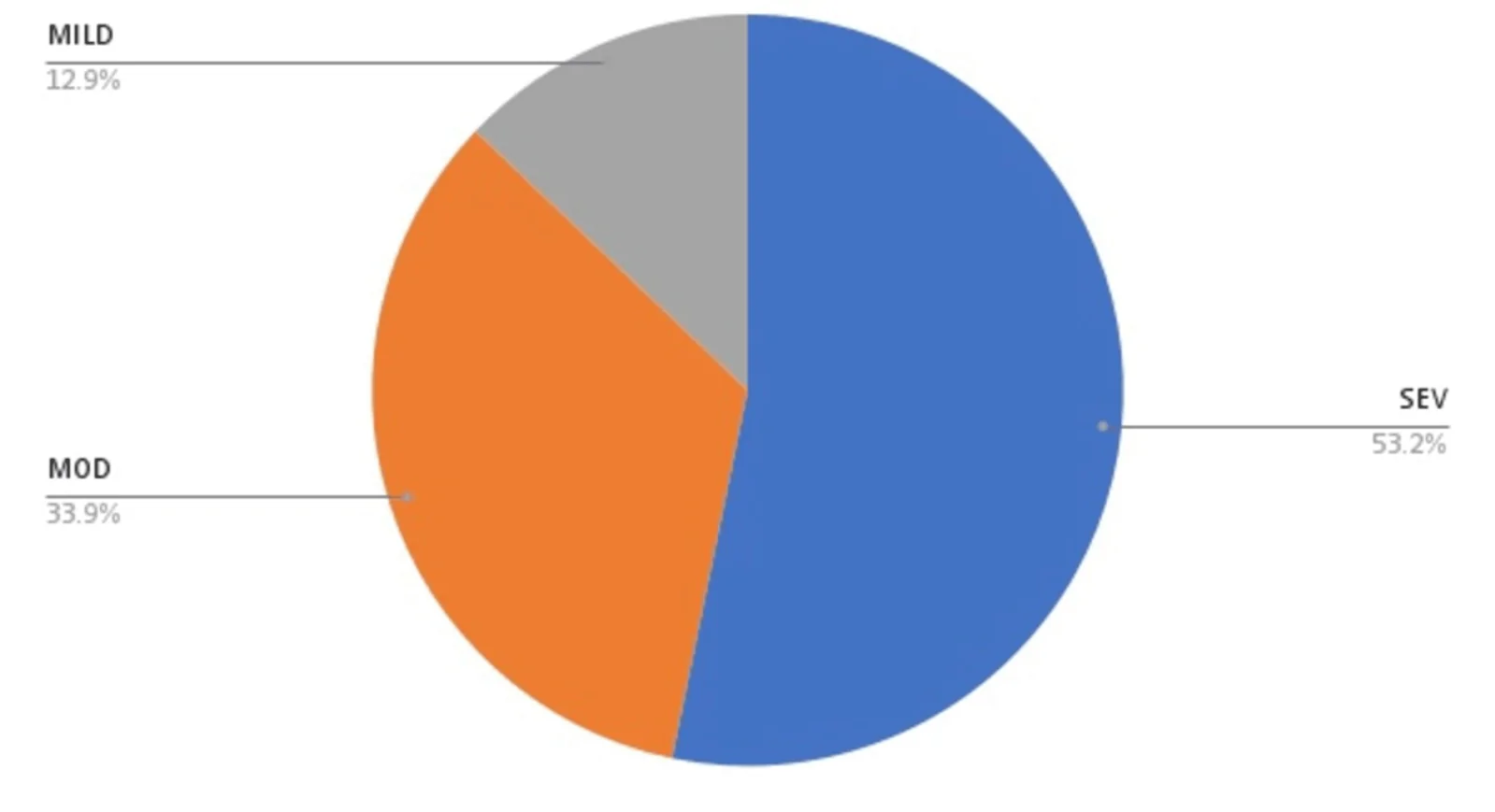
Clinical dysfunction distribution at first visit MOD: moderate; SEV: severe

At six weeks (Figure 2), severe dysfunction was no longer observed, while the proportion of patients with mild dysfunction increased markedly to 75.8% (n=48), suggesting substantial early functional recovery.

**Figure 2:**
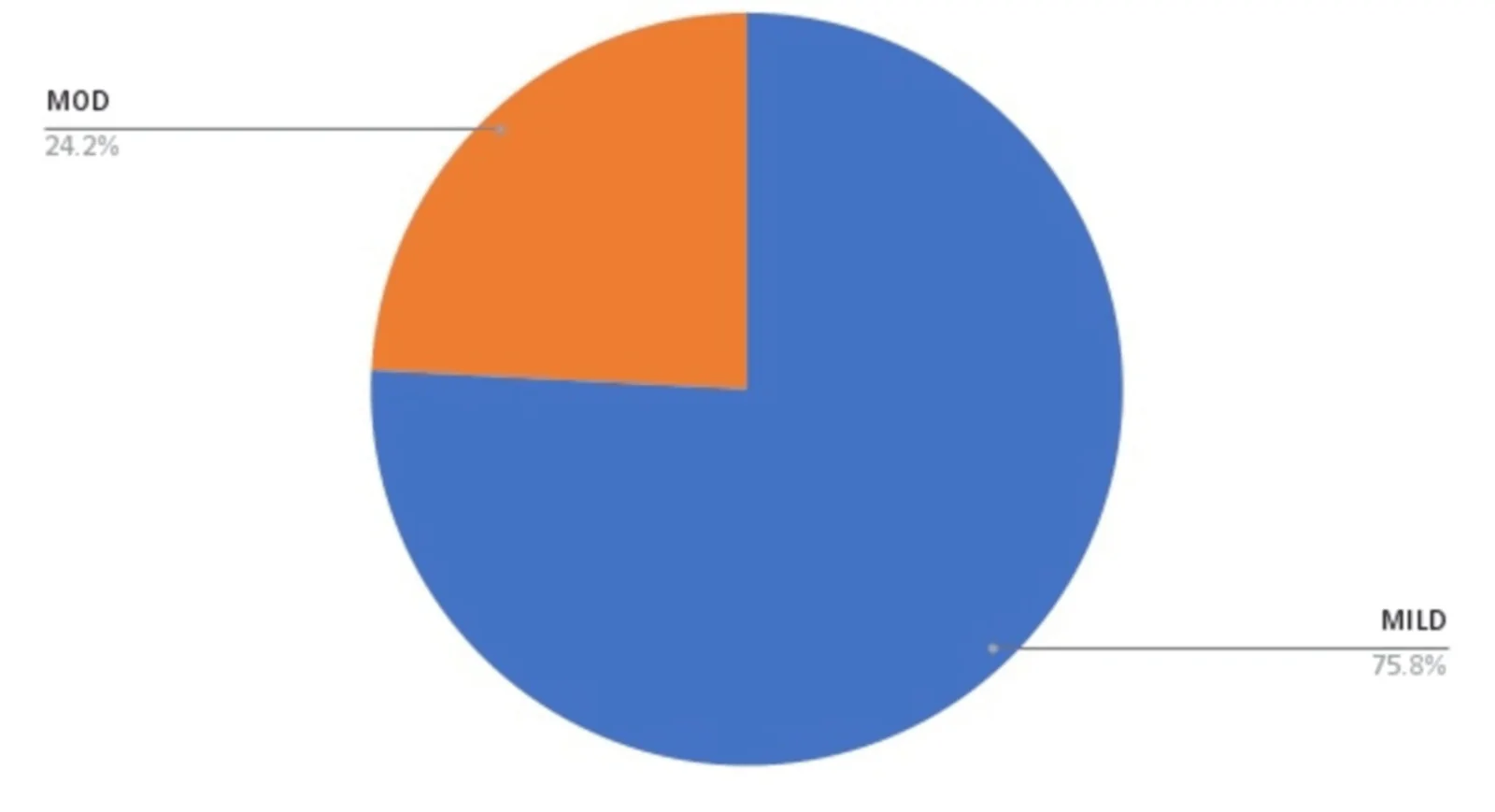
Clinical dysfunction distribution at six weeks MOD: moderate

By three months (Figure 3), moderate and severe dysfunction were completely absent, with 41.9 % (n=26) of patients demonstrating mild dysfunction and 58.1% (n=37) demonstrating no clinical dysfunction.

**Figure 3:**
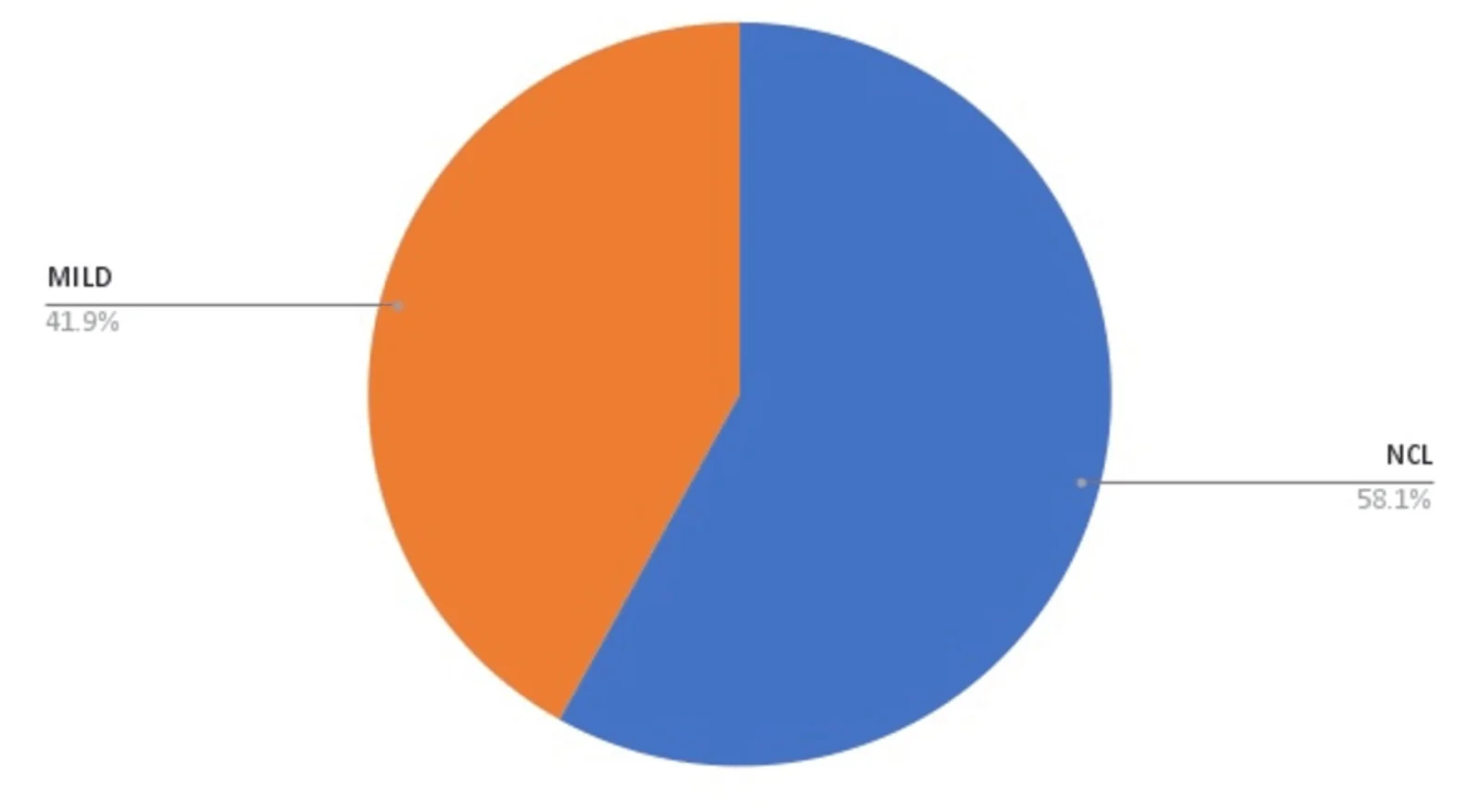
Clinical dysfunction distribution at third month visit NCL: no clinical limitation

Overall, the trajectory indicates substantial recovery of temporomandibular joint function over the three-month follow-up period, with the greatest redistribution occurring during the initial six weeks and continued improvement thereafter (Figure 4).

**Figure 4:**
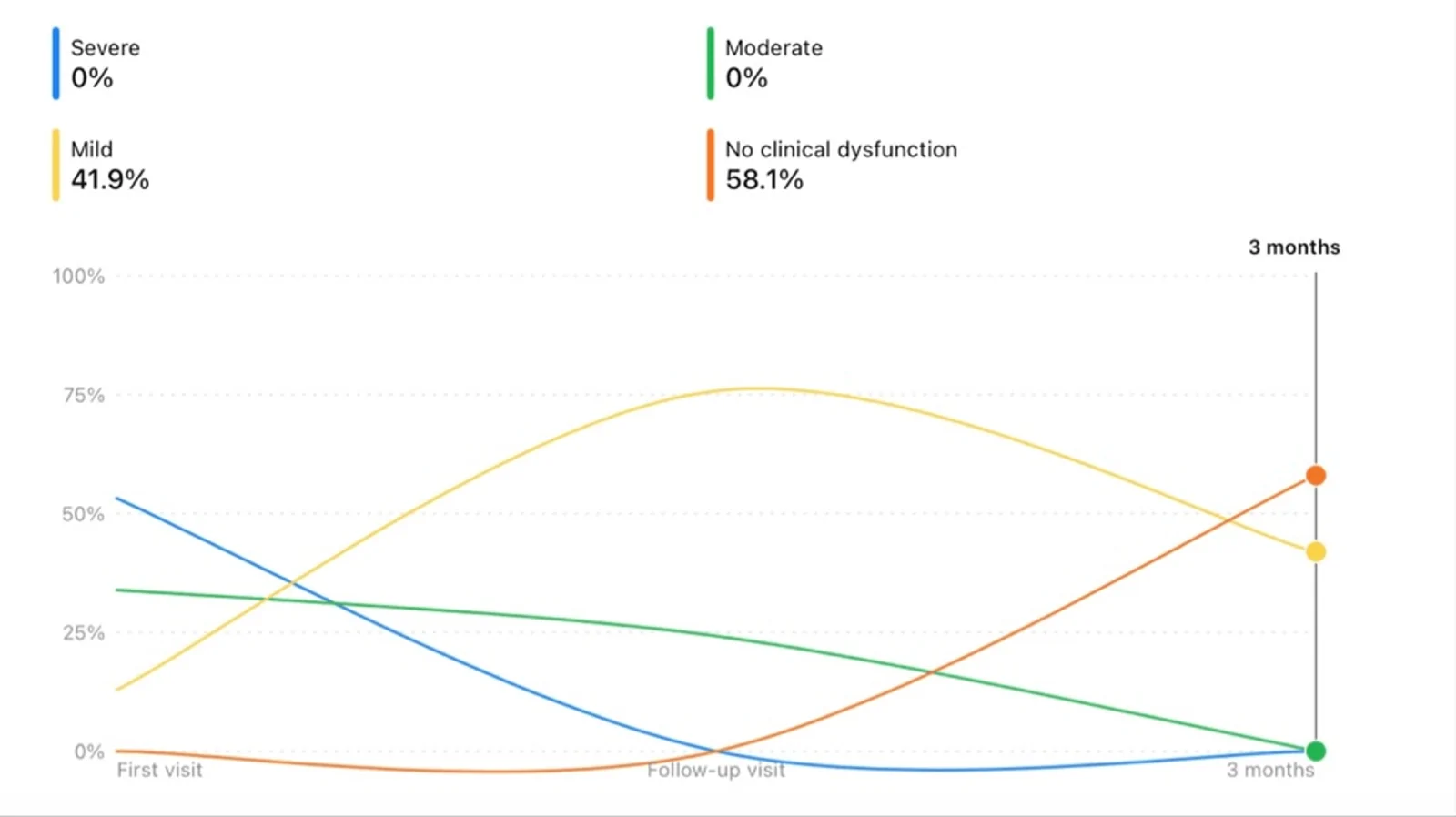
Clinical dysfunction trajectory across first week to third month visit

Mandibular mobility recovery

Baseline mobility restriction showed a mean score of 2.14 +/- 1.82, which significantly decreased to 1.13 +/- 0.71 at six weeks and reached near-complete recovery (0.25 +/- 0.44, median 0.0) at the three-month follow-up (p < 0.001). This confirms progressive return toward uncompromised mandibular movement (Figure 5).

**Figure 5:**
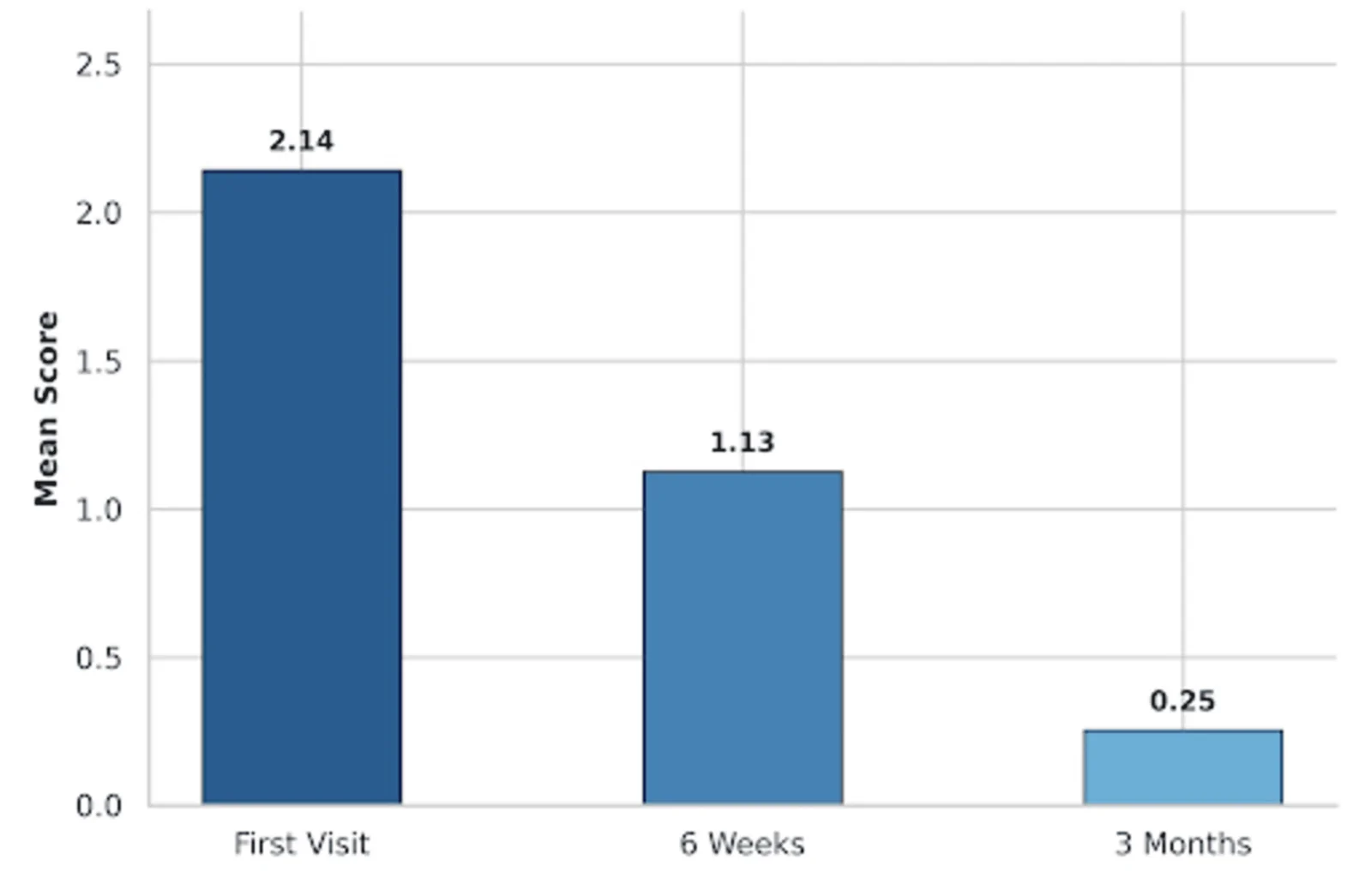
Progression of mean mandibular mobility score across visits

Overall clinical burden

The total cumulative symptom score dropped markedly from a baseline of 11.73 +/- 5.94 to 4.95 +/- 1.72 at six weeks, reaching a minimal residual score of 1.11 +/- 1.63 at 3 months, highlighting highly favorable long-term recovery outcomes across all evaluated cases (Figure 6). 

**Figure 6:**
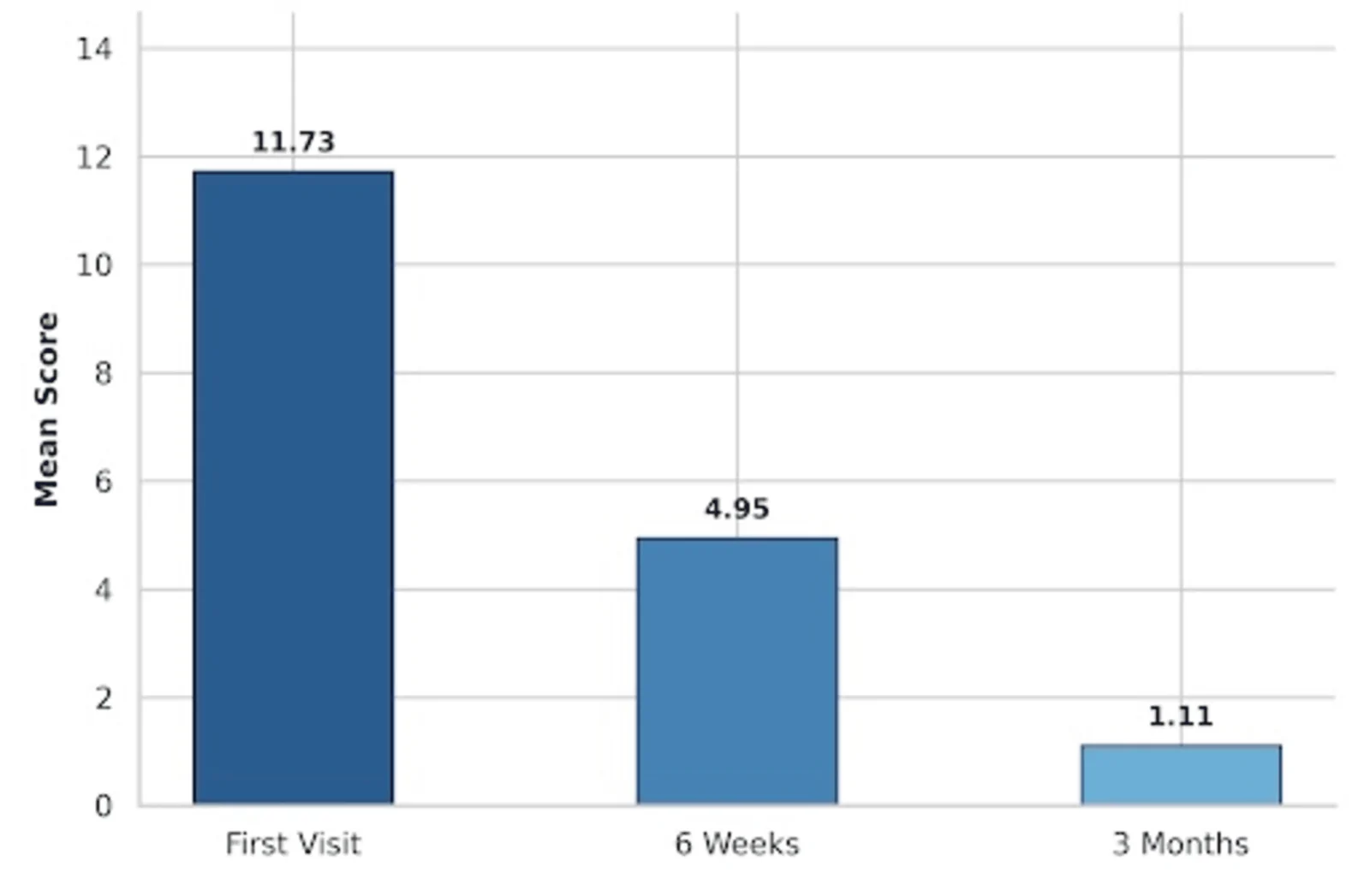
Progression of mean total cumulative scores across visits

Pain parameters and TMJ function

Pain in mandibular movement dropped sharply from a baseline mean of 3.54 +/- 1.94 to 1.00 +/- 0.00 at six weeks, further declining to 0.22 +/- 0.42 at three months (Table 4).

**Table 4:** Comprehensive descriptive and comparative statistics table TMJ: temporomandibular joint; IQR: interquartile range

Clinical parameter	First visit (Mean +/- SD/Median (IQR))	Six-week follow-up (Mean +/- SD/Median (IQR))	Three-month follow-up (Mean +/- SD Median (IQR))	Friedman test (p-value)
Mandibular mobility	2.14 +/- 1.82	1.13 +/- 0.71	0.25 +/- 0.44	< 0.0001 (p = 6.31 \times 10^{-23})
Impaired TM joint	3.35 +/- 1.99	1.00 +/- 0.00	0.22 +/- 0.42	< 0.0001 (p = 5.77 \times 10^{-24})
Muscle pain	0.43 +/- 0.50	0.00 +/- 0.00	0.00 +/- 0.00	< 0.0001 (p = 1.88 \times 10^{-12})
TMJ pain	2.27 +/- 1.88	1.83 +/- 1.63	0.41 +/- 0.50	< 0.0001 (p = 6.46 \times 10^{-21})
Pain in mandibular movement	3.54 +/- 1.94	1.00 +/- 0.00	0.22 +/- 0.42	< 0.0001 (p = 3.42 \times 10^{-24})
Total cumulative score	11.73 +/- 5.94	4.95 +/- 1.72	1.11 +/- 1.63	< 0.0001 (p = 9.64 \times 10^{-27})

TMJ pain scores demonstrated a steady downward trajectory from 2.27 +/- 1.88 pre-intervention to 1.83 +/- 1.63 at six weeks, dropping to 0.41 +/- 0.50 at three months (Figure 7).

**Figure 7:**
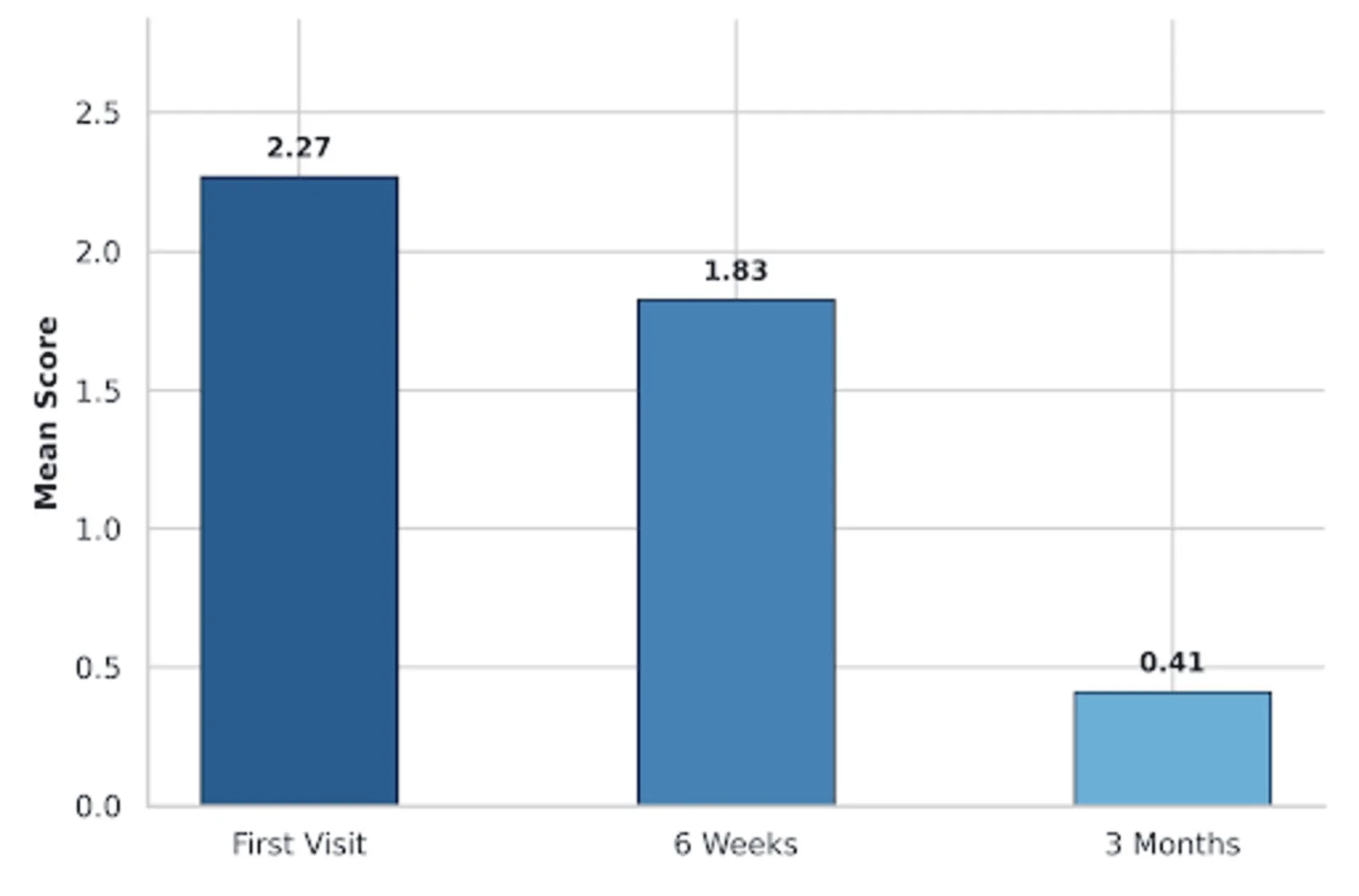
Progression of mean TMJ pain score across visits TMJ: temporomandibular joint

Muscle pain resolved completely across the cohort by the six-week and three-month marks (mean 0.00) (Figure 8).

**Figure 8:**
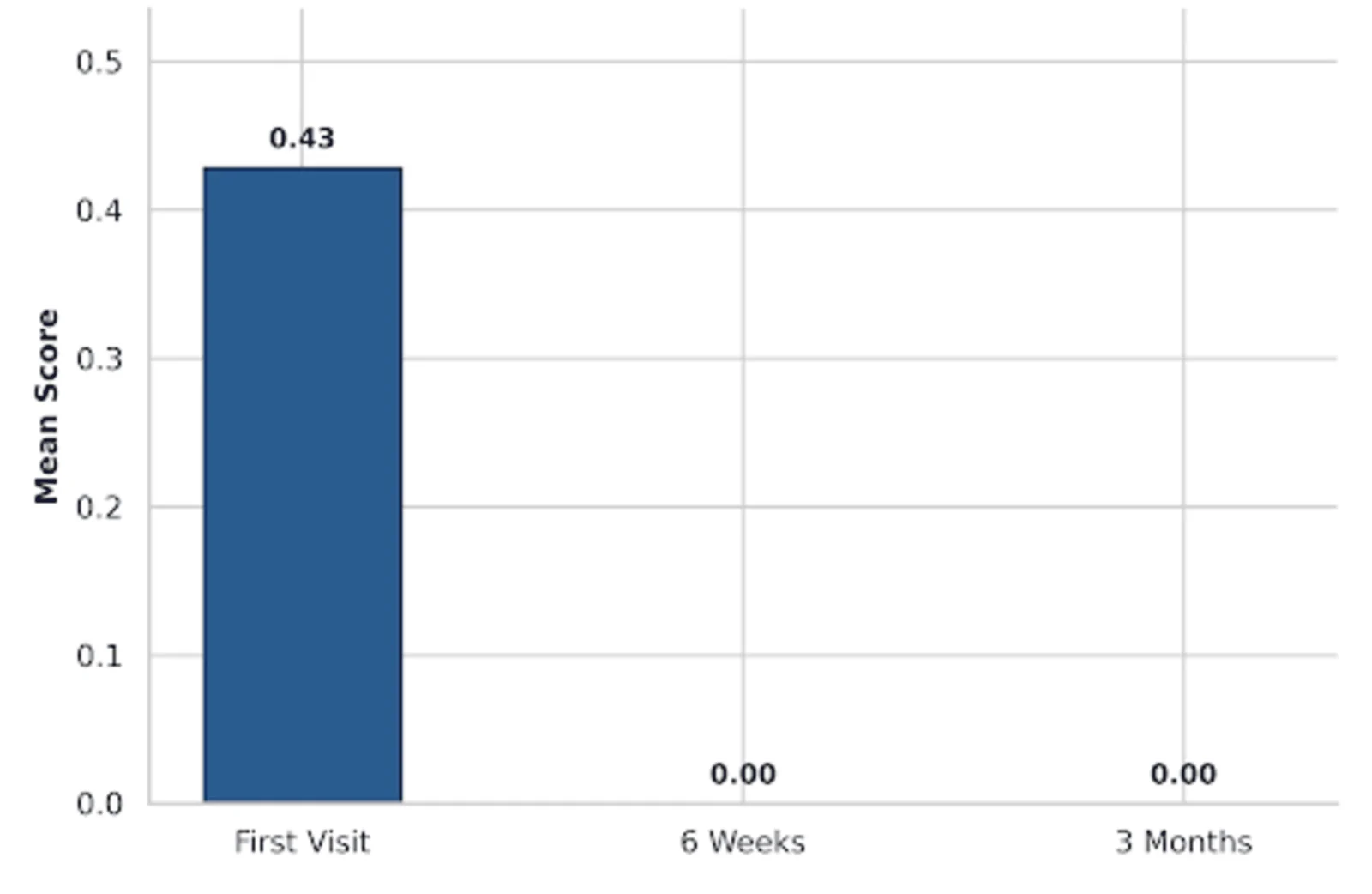
Progression of mean muscle pain score across visits

## Discussion

The longitudinal assessment in the present study provides a more meaningful representation of recovery than a single postoperative assessment. Following condylar fracture, impairment of mandibular function may result from several interacting factors, including pain, joint effusion, edema, muscular spasm, altered condylar position, disruption of the normal occlusal relationship and temporary restriction of mandibular movements [7]. Progressive improvement in clinical dysfunction over subsequent visits may therefore reflect the combined effects of fracture healing, resolution of inflammation and pain, restoration of neuromuscular coordination and gradual adaptation of the TMJ. The trajectory observed in the present study should therefore be interpreted as a dynamic process of adaptation and recovery.

The use of Helkimo’s Clinical Dysfunction Index in the present study provides a standardized clinical approach to evaluating this functional recovery. The index incorporates clinically relevant parameters such as limitation of mandibular movement, impaired TMJ function, muscle tenderness, TMJ tenderness and pain associated with mandibular movement. Thus, changes in the clinical dysfunction score across follow-up visits can be interpreted as an overall measure of functional recovery rather than as an isolated post operative assessment of fracture healing.

The improvement in mandibular movement over time is particularly important because condylar fractures can alter the normal biomechanics of the mandible [8-10]. During mouth opening and other movements of the condyle , translation and rotation of the condyle normally occur in a coordinated manner. Following fracture, temporary alteration of condylar position and movement may produce deviation or limitation of opening. With conservative treatment, functional adaptation of the masticatory muscles and remodeling of the injured condylar region may contribute to restoration of mandibular movement. Progressive mandibular mobilization and functional exercises are important for preventing prolonged restriction of movement and encouraging neuromuscular adaptation. The observed improvement in dysfunction scores across follow-up visits reflects not only biological healing but also the patient’s progressive return to normal mandibular function. However, this study is a single arm observational design with absence of comparison group which limits the ability to attribute the observed improvement specifically to the followed treatment protocol.

The present study demonstrates a progressive reduction in Helkimo dysfunction scores across the three visits, suggesting a favorable functional trajectory following closed management. The observed data demonstrate temporal improvement in TMJ function following conservative treatment with intermittent mobilization but due to the observational design and absence of a control or comparison group, the observed improvement in functional parameters cannot be interpreted as evidence of treatment effectiveness or causality.The magnitude of improvement between successive visits also provides insight into the phase of recovery, with greater improvement during the earlier follow-up period potentially representing resolution of acute post-traumatic symptoms and later improvement reflecting progressive remodeling and neuromuscular adaptation [11,12].

Conversely, patients who demonstrate persistent or minimal improvement in dysfunction scores should not be interpreted solely on the basis of the overall group trend. Individual variation in recovery may be influenced by factors such as fracture level, displacement, unilateral or bilateral involvement, associated mandibular fractures, age, initial limitation of movement, adherence to physiotherapy and the presence of pre-existing TMJ dysfunction [13-15]. These factors may explain why apparently similar fractures can demonstrate different functional recovery trajectories.

The findings also reinforce the importance of serial clinical assessment after conservative treatment of condylar fractures. A single follow-up examination may underestimate the eventual recovery potential of the TMJ or, alternatively, may fail to identify patients whose dysfunction persists. Longitudinal monitoring using a reproducible clinical index can therefore provide a practical means of identifying the direction and rate of functional recovery.An important advantage of the present longitudinal design is that it allows each patient to serve, to some extent, as their own functional reference over time. Rather than categorizing the outcome simply as “successful” or “unsuccessful,” serial Helkimo assessments demonstrate whether the patient follows a favorable, delayed or plateauing recovery trajectory. This approach is particularly relevant for condylar fractures because functional recovery may continue beyond the period in which the fracture itself appears clinically stable. 

The study should, however, be interpreted in light of certain limitations. Helkimo’s Clinical Dysfunction Index is a clinical assessment tool and does not directly quantify condylar morphology or internal TMJ pathology [16]. Functional scores may also be influenced by subjective symptoms such as pain and tenderness. Furthermore, the trajectory observed over the study’s follow-up period may not represent the complete long-term course of TMJ adaptation, as condylar remodeling and neuromuscular adaptation can continue for an extended period. Although the same examiner performed all assessments using a standardized protocol, formal examiner calibration and inter or intra-examiner reliability testing were not undertaken, which represents a methodological limitation. Larger studies with longer follow-up and correlation with radiographic or imaging findings would therefore provide a more comprehensive understanding of the relationship between structural remodeling and functional recovery.

Overall, the present study emphasizes that the outcome of conservatively managed condylar fractures should be viewed as a functional trajectory rather than a single endpoint. Progressive improvement in mandibular function and reduction in clinical TMJ dysfunction over successive visits would indicate that the TMJ undergoes substantial functional adaptation following closed treatment. Serial assessment using Helkimo’s Clinical Dysfunction Index can therefore serve as a useful clinical method for documenting this recovery and identifying patients who require closer monitoring or additional intervention.

## Conclusions

The central clinical implication of the present study is that successful management of a condylar fracture should be judged by the recovery of TM joint function over time. Regular assessment of mouth opening, mandibular excursions, TMJ and muscle tenderness, pain and overall dysfunction can help clinicians determine whether the patient is following an expected recovery trajectory and can facilitate early identification of persistent functional impairment. Although the study demonstrates progressive improvement in mandibular functional status during the followup period, the observational design with lack of comparison group does not permit attribution of these changes specifically to treatment protocol and establishment of causality.
